# Impact of blue light on skin pigmentation in patients with melasma

**DOI:** 10.1111/srt.13401

**Published:** 2023-07-02

**Authors:** Lingjia Li, Xin Jiang, Yin Tu, Yaqi Yang, Xin Zhang, Hua Gu, Li He

**Affiliations:** ^1^ Department of Dermatology Jiangnan University Medical Center, Jiangnan University Jiangsu China; ^2^ Department of Dermatology The First Affiliated Hospital of Kunming Medical University Kunming Medical University Yunnan China

**Keywords:** healthy individuals, melasma, observational study, skin pigmentation, visible light

## Abstract

**Background:**

The difference in skin pigmentation induced by blue light between melasma patients and healthy people has not been reported. This study aimed to explore the impact of different doses of blue light irradiation on the pigmentation of the skin of non‐exposed areas in female melasma patients with III‐IV‐type skin and healthy women.

**Materials and methods:**

This observational study enrolled patients with melasma and healthy people at the First Affiliated Hospital of Kunming Medical University between January and April 2021. The outcomes were the degree of pigmentation, ΔL*, and ΔITA* values.

**Results:**

Forty‐two (21/group) participants were enrolled. After irradiation with different doses of blue light, different degrees of pigmentation could be observed in the irradiated area of the skin of female melasma patients and healthy women. The △L* and △ITA* values in the irradiated area of the skin of healthy women were higher than in female melasma patients after blue light irradiation at 20 J/cm^2^ (*p* < 0.05). There were no significant differences in the pigmentation scores, △L* values, and △ITA* values in the irradiated areas of skin at different time points after irradiation with the other doses of blue light (*p* > 0.05).

**Conclusion:**

Blue light at 20 J/cm^2^ induced a smaller change in pigmentation in melasma patients than in healthy women, but the effect of blue light at 40–80 J/cm^2^ was similar.

## 1 INTRODUCTION

Melasma is a common acquired skin pigmentation disease characterized by light‐brown to dark‐brown pigmented spots on the exposed areas of the face, such as the forehead, cheeks, and lower jaw, and is more common in darker‐skin III‐IV‐type Asian or Hispanic women.^1^, ^2^, ^3^, ^4^ The pathogenesis of melasma is not yet clear, and it is believed to be mostly related to solar radiation, genetics, and sex hormone levels.^2^, ^5^, ^6^, ^7^ Solar radiation is one of the important causes of melasma.^1^ Melasma may be associated with photoaging and solar elastosis and tends to be a chronic condition after treatment.^2^, ^3^

Sunlight is divided into extreme ultraviolet (UV) rays, UV rays, visible light, and infrared rays according to the different wavelengths.^8^ UV irradiation can induce melanin synthesis, cause pigmentation in the irradiated area, and induce pigmentation diseases, including melasma.^1^, ^9^ Visible light (400–700 nm) accounts for about 50% of solar radiation.^10^ Visible light, particularly high‐energy visible light (400–500 nm), also called blue light, participates in skin aging.^11^, ^12^ Blue light can cause skin hyperpigmentation, particularly in dark‐skinned people.^13^ The degree of pigmentation is more obvious than the pigmentation induced by UV rays, and the blue light‐induced pigmentation can last as long as 3 months.^13^, ^14^ Blue light can induce immediate melanosis, continuous melanosis, and delayed melanosis.^13^, ^14^ These effects can be observed in all skin types, including in patients with pigmentation diseases.^10^, ^15^

The reason for this effect of blue light is that melanocyte opsin 3 activates calmodulin‐dependent protein kinase II, transcription factor cAMP‐responsive element‐binding protein, mitogen‐activated protein kinase P38, and extracellular regulated protein kinase by sensing external blue light and induces melanin synthesis.^16^ In ex vivo skin specimens, gene expression and tyrosinase activity are increased by visible light.^17^ Persistent pigment darkening could last up to 10 days when repeated exposures were performed, but pigmentation faded after 24 h when a single exposure was used.^17^ Visible light also has effects on the skin barrier function.^18^

It could be hypothesized that healthy people will have a more serious degree of melanosis after exposure to blue light than normal people because of lower baseline melanosis. Therefore, this study aimed to explore the impact of blue light irradiation on the pigmentation in the skin of non‐exposed areas in female melasma patients with III‐IV‐type skin and healthy women.

## 2 MATERIALS AND METHODS

### 2.1 Study design and population

This observational study included patients with melasma and healthy people at the First Affiliated Hospital of Kunming Medical University between January and April 2021. This study was approved by the Ethics Committee of the First Affiliated Hospital of Kunming Medical University. Informed consent was obtained from all subjects, and the informed consent form was signed. This study was performed following the tenets of the Declaration of Helsinki.

The inclusion criteria were 1) diagnosis of melasma, 2) 20–50 years old, 3) not pregnant or breastfeeding, 4) living in Yunnan for ≥3 years, and 5) no folliculitis, atopic dermatitis, port‐wine stains, etc., in the irradiated area that could affect the assessments in the irradiated area. The exclusion criteria were 1) pigmentation diseases on the face, 2) history of immune system diseases, photosensitivity diseases, inflammatory skin diseases, or major organ diseases, or 3) having taken photosensitive drugs recently.

For the healthy women group, age‐matched individuals were selected. The selection criteria were the same as for the patients, except for the melasma diagnosis.

### 2.2 Procedure

Blue light with a wavelength of 400–520 nm was produced by a blue light irradiator (Sellamed VIS 400, Sellas, Germany). The average blue light irradiance intensity at a distance of 10 cm from the light source was measured using a blue light radiometer (FL‐1D, Beijing Normal University Photoelectric Instrument Factory, Beijing, China) and was 50 mW/cm^2^. A 1‐cm‐thick thermal insulation PVC board was used to make a cuboid mold with a length of 32.6 cm, a width of 24.6 cm, and a height of 10 cm. The inside was painted black. There were two rows of irradiation holes on the front, 4 cm from top to bottom and 3 cm from left to right, and there were two square irradiating holes with an area of 1 cm^2^ in each row. All participants were exposed to blue light irradiation, with the back slightly touching the surface of the mold. Blue light at 20, 40, 60, and 80 J/cm^2^ was used to irradiate the skin on the back of the participants. The irradiated areas were photographed at 0 h, 24 h, 1 week, and 2 weeks after irradiation.

### 2.3 Outcomes

The pigmentation degree in the irradiated area was assessed according to the Investigator's Global Assessment Scale (IGAS) (Table S1).^19^ The irradiation intensity of the blue light machine was 50 ± 5 mW/cm^2^. After irradiation, the irradiation site was marked. The values were measured at the same irradiation site at different time points, and then the IGAS was calculated. A portable spectrophotometer (CM‐2600d; Konica Minolta, Japan) was used to measure the L* values and b* values of the irradiated and non‐irradiated areas at 0 h, 24 h, 1 week, and 2 weeks after irradiation, and the corresponding ΔL* value and ΔITA value were calculated. The L* value represents brightness. ΔL* value = L* value of irradiated area‐ L* value of non‐irradiated area. The larger the ΔL* value is, the more obvious the pigmentation is. ITA value = [arctan(L*−50)/b*]x180/π. ΔITA value = ITA value at the irradiated area‐ ITA value at the non‐irradiated area. The larger the ΔITA value is, the more obvious the pigmentation is.^19^, ^20^

### 2.4 Statistical analysis

SPSS 26.0 (IBM Corp., Armonk, NY, USA) was used for data analysis. The continuous data were presented as means ± standard deviation and analyzed using repeated‐measures ANOVA to compare the data within a given group among time points and Student's t‐test to compare groups. *p* < 0.05 was statistically significant.

## 3 RESULTS

Forty‐two (21/group) female patients with melasma and healthy women (all with III‐IV skin phototype and living in Yunnan) were enrolled. There were no differences in age (35.4 ± 1.9 vs. 35.8 ± 1.9 years, *p* = 0.83), L* (65.46 ± 3.01 vs. 63.82 ± 4.02, *p* = 0.15), and ITA (32.36 ± 6.87 vs. 28.66 ± 9.34, *p* = 0.17) between the two groups (Table 1).

**TABLE 1:** Baseline characteristics of melasma patients and healthy women.

	Melasma patients (*n* = 21)	Healthy women (*n* = 21)	*p*
Age	35.76 ± 8.84	35.38 ± 8.71	0.83
L*	65.46 ± 3.01	63.82 ± 4.02	0.15
ITA	32.36 ± 6.87	28.66 ± 9.34	0.17

**FIGURE 1:**
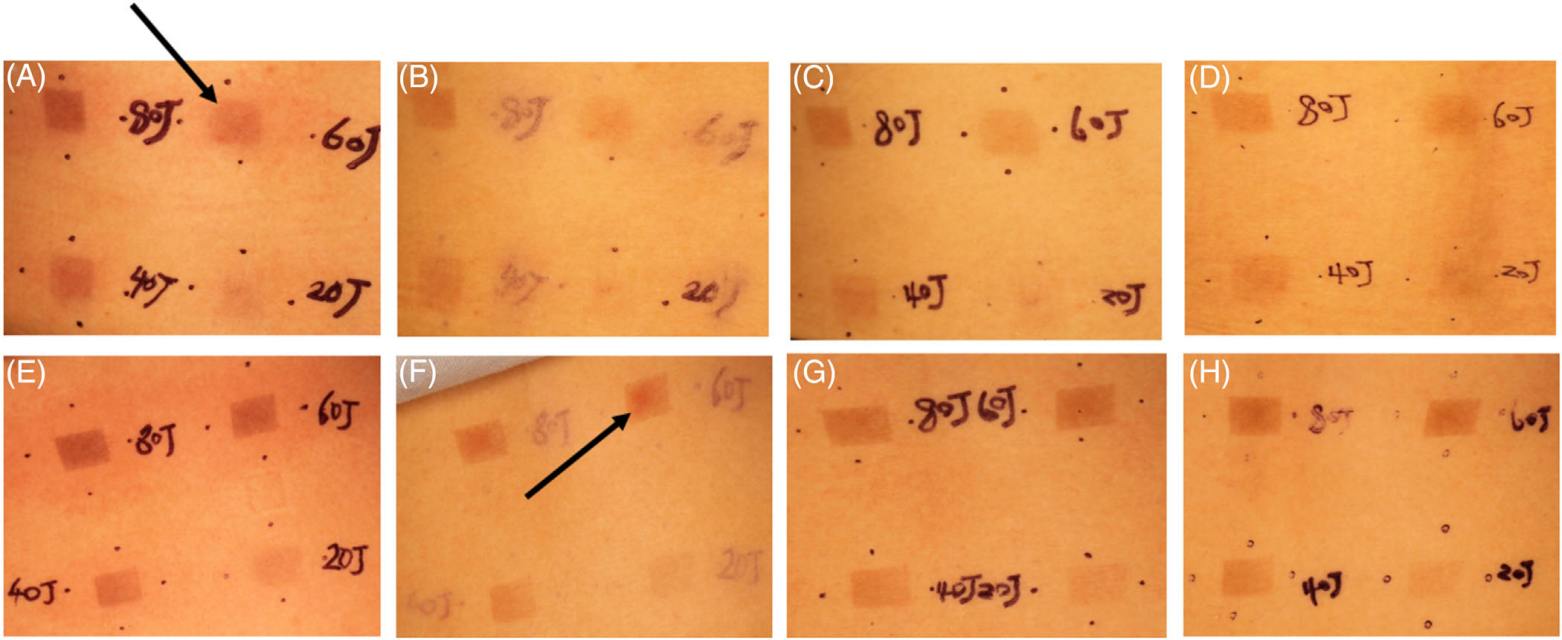
(Upper panel) Pigmentation in the skin on the back of melasma patients induced by blue light at (a) 0 h, (b) 24 h, (c) 1 week, and (d) 2 weeks. (Bottom panel) Pigmentation in the skin on the back of healthy women was induced by blue light at (e) 0 h, (f) 24 h, (g) 1 week, and (h) 2 weeks. The blue arrows indicate transient erythema.

Blue light irradiation of 20, 40, 60, and 80 J/cm^2^ could induce different degrees of pigmentation in the skin on the back of melasma patients and healthy women, and the degree of pigmentation was dose‐dependent. A dose as low as 20 J/cm^2^ of blue light irradiation could induce pigmentation in the skin of melasma patients and healthy women, which did not completely subside after 2 weeks. In addition, it was also observed that after blue light irradiation, transient erythema also appeared in the irradiated area, which subsided within 24 h (Figure 1).

The IGA scores on the skin of the non‐exposed areas in the melasma patient and healthy women groups at 0 h of irradiation of blue light at 20, 40, 60, and 80 J/cm^2^ were higher than those at 24 h, 1 week, and 2 weeks. The IGAS were not significantly different between the two groups, and there were no time effects after 20, 49, and 80 J/cm^2^ blue light irradiation were all lower than those of the healthy women group, but the melasma patients had higher IGAS after 60 J/cm^2^ blue light irradiation, with significant changes in time, but no time × group interaction was observed (Figure S1 and Table 2).

**TABLE 2:** Pigmentation IGA scores of the skin in the irradiated areas in the melasma patient and healthy women groups at different time points.

Blue light intensity	Time	Melasma patients (*n* = 21)	Healthy women (*n* = 21)	*P*_Time_	*P*_Group_	*P*_Interaction_
20 J/cm^2^	0 h	0.83 ± 0.09	0.98 ± 0.16	ref	0.068	0.503
	24 h	0.67 ± 0.09	0.81 ± 0.11	0.232		0.209
	1 week	0.64 ± 0.10	0.83 ± 0.14	0.227		0.282
	2 weeks	0.67 ± 0.11	0.81 ± 0.13	0.254		0.480
40 J/cm^2^	0 h	2.05 ± 0.22	1.67 ± 0.22	ref	0.073	0.233
	24 h	1.69 ± 0.17	1.40 ± 0.14	0.079		0.300
	1 week	1.55 ± 0.15	1.50 ± 0.15	0.073		0.801
	2 weeks	1.67 ± 0.17	1.40 ± 0.16	0.096		0.304
60 J/cm^2^	0 h	2.74 ± 0.24	2.14 ± 0.24	ref	0.023	0.055
	24 h	2.00 ± 0.15	1.90 ± 0.17	0.02		0.531
	1 week	2.00 ± 0.15	1.90 ± 0.16	0.018		0.328
	2 weeks	2.05 ± 0.18	1.83 ± 0.20	0.025		0.424
80 J/cm^2^	0 h	2.88 ± 0.32	2.29 ± 0.26	ref	0.240	0.219
	24 h	2.12 ± 0.18	2.07 ± 0.16	0.120		0.896
	1 week	2.14 ± 0.23	1.98 ± 0.18	0.109		0.571
	2 weeks	2.12 ± 0.24	1.95 ± 0.19	0.085		0.582

The mean values of ΔL* and ΔITA* on the skin of the non‐exposed areas in the melasma patient and healthy women groups at 0 h with 20, 40, 60, and 80 J/cm^2^ blue light irradiation were higher than those at 24 h, 1 week, and 2 weeks (all *p* < 0.01).

The mean values of ΔL* and ΔITA* of the melasma patient group at different time points were lower than those of the healthy women group after 20 J/cm^2^ blue light irradiation (all *p* > 0.05) (Figures S2 and S3 and Table 3).

**TABLE 3:** △L* and △ITA values at different time points.

Indicators	Blue light intensity	Time	Melasma patients (*n* = 21)	Healthy women (*n* = 21)	*P*_Time_	*P*_Group_	*P*_Interaction_
△L*	20 J/cm^2^	0 h	−7.40 ± 0.75	−8.11 ± 0.93	ref	0.029	0.558
		24 h	−4.77 ± 0.51	−5.94 ± 0.63	0.001		0.166
		1 week	−4.08 ± 0.53	−5.52 ± 0.75	<0.001		0.187
		2 weeks	−3.66 ± 0.49	−5.65 ± 0.71	<0.001		0.038
	40 J/cm^2^	0 h	−10.55 ± 0.86	−10.93 ± 0.97	ref	0.574	0.772
		24 h	−7.22 ± 0.61	−7.24 ± 0.69	<0.001		0.980
		1 week	−6.32 ± 0.69	−6.96 ± 0.67	<0.001		0.538
		2 weeks	−6.01 ± 0.73	−6.81 ± 0.67	<0.001		0.474
	60 J/cm^2^	0 h	−11.55 ± 0.65	−12.16 ± 0.83	ref	0.603	0.564
		24 h	−7.82 ± 0.66	−7.66 ± 0.67	<0.001		0.867
		1 week	−6.94 ± 0.73	−7.63 ± 0.81	<0.001		0.526
		2 weeks	−6.26 ± 0.58	−7.34 ± 0.75	<0.001		0.660
	80 J/cm^2^	0 h	−12.04 ± 0.95	−12.07 ± 1.07	ref	0.445	0.980
		24 h	−8.25 ± 0.73	−8.77 ± 0.83	<0.001		0.638
		1 week	−6.96 ± 0.67	−7.79 ± 0.80	<0.001		0.431
		2 weeks	−6.85 ± 0.71	−7.72 ± 0.79	<0.001		0.418
△ITA*	20 J/cm^2^	0 h	−13.66 ± 1.93	−14.29 ± 2.19	ref	0.033	0.833
		24 h	−7.75 ± 1.16	−10.33 ± 1.47	0.006		0.176
		1 week	−7.56 ± 1.16	−10.86 ± 1.52	0.008		0.148
		2 weeks	−7.24 ± 1.10	−11.12 ± 1.43	0.007		0.037
	40 J/cm^2^	0 h	−20.39 ± 2.19	−20.87 ± 2.41	ref	0.574	0.881
		24 h	−12.82 ± 1.41	−13.48 ± 1.53	<0.001		0.752
		1 week	−12.45 ± 1.45	−14.05 ± 1.38	<0.001		0.538
		2 weeks	−12.32 ± 1.57	−13.84 ± 1.35	<0.001		0.468
	60 J/cm^2^	0 h	−22.62 ± 1.74	−23.64 ± 2.02	ref	0.509	0.705
		24 h	−14.05 ± 1.53	−14.36 ± 1.42	<0.001		0.880
		1 week	−13.68 ± 1.60	−15.31 ± 1.71	<0.001		0.492
		2 weeks	−12.48 ± 1.40	−14.71 ± 1.51	<0.001		0.642
	80 J/cm^2^	0 h	−23.76 ± 2.51	−23.58 ± 2.71	ref	0.426	0.961
		24 h	−14.86 ± 1.73	−16.37 ± 1.96	0.001		0.567
		1 week	−13.69 ± 1.42	−15.99 ± 1.66	<0.001		0.300
		2 weeks	−13.46 ± 1.66	−15.86 ± 1.98	<0.001		0.303

## 4 DISCUSSION

This study suggested that blue light at 20 J/cm^2^ induced a smaller change in pigmentation in melasma patients than in healthy women, but the effect of blue light at 40, 60, and 80 J/cm^2^ was similar.

Consistent with previous reports, in the present study, blue light could induce pigmentation in III‐IV‐type skin, and the degree of pigmentation was dose‐dependent.^11^, ^12^, ^13^ The reason is that opsin 3 in melanocytes senses the stimulation from external blue light, induces an increase in melanin synthesis through a series of signaling cascades pathways, and blue light can also induce melanocytes in type III and IV skin to form the tyrosinase and dopa isomerase protein complex, which ultimately leads to the formation of long‐lasting pigmentation.^13^, ^16^ Different from Mahmoud et al.,^14^ it was observed in this study that 20‐J/cm^2^ blue light irradiation could induce obvious pigmentation in III‐IV‐type skin, and it lasted for a long time, suggesting that blue light within the visible light is the main light source to induce skin pigmentation, even at low levels. In addition, a transient erythematous reaction was also observed in the irradiated area, which could be caused by skin telangiectasis caused by the photothermal effect.^13^, ^14^ In the present study, the changes in L* in ITA* were smaller in melasma patients than in healthy individuals, suggesting that the baseline skin pigmentation mechanisms were already active and that low‐dose blue light (20 J/cm^2^) had a small activating effect. Supporting these results, Duteil et al.^21^ reported that short‐term repeated exposure to blue light irradiation from electronic devices in melasma patients did not deepen the pigmentation of skin lesions, suggesting that repeated low‐dose blue light irradiation has a limited effect on the pigmentation of the skin in melasma patients; it could be hypothesized that the activating effect of blue light from electronic devices is below the baseline melanosis level in melasma patients. On the other hand, higher doses of blue light (40, 60, and 80 J/cm^2^) had similar effects in patients with melasma and healthy controls.

In this study, melasma patients and healthy women were exposed to a single dose of 20–80 J/cm^2^ blue light irradiation, and there were no significant differences in the IGAS at the irradiation area between groups, suggesting that melasma patients have similar sensitivity to pigmentation induced by blue light than healthy women, that is, a single small‐dose of blue light irradiation has limited effects on melasma patients. Blue light can induce immediate melanosis, continuous melanosis, and delayed melanosis.^13^, ^14^ In the present study, melanosis was immediate in both groups and was continuous over 2 weeks, but no increase or flare‐up in melanosis was observed, indicating the absence of delayed melanosis, at least over 2 weeks. Still, this study did not examine the effect of repeated or long‐duration exposures. Indeed, people working all day long with computers are continuously exposed to blue light several hours a day in addition to the exposure from smartphones and tablets.

Besides occupational exposure, blue light in daily life mainly comes from sunlight. The solar radiation intensity on the earth's surface is 440 W/m^2^; the blue light energy accounts for one‐fifth of the visible light, and the blue light radiation intensity is about 8.8 mW/cm^2^.^13^ In the present study, a blue light radiometer was used to continuously measure the blue light irradiation intensity at local noontime for 1 week. The average blue light irradiation intensity was 8.1 mW/cm^2^, and the 20–80 J/cm^2^ blue light irradiation dose is equivalent to sunlight exposure for 42–165 min. Therefore, it suggests that the blue light irradiation dose accumulated during a sunlight exposure of < 165 min does not aggravate pigmentation in melasma patients. Nevertheless, in addition to blue light, sunlight also includes ultraviolet rays, which can synergistically induce pigmentation, and broad‐spectrum sunscreens that protect against ultraviolet rays and blue light can effectively reduce the recurrence rate of melasma patients.^19^, ^22^ Therefore, we recommend that melasma patients still take appropriate sun protection measures, including umbrellas, wearing long clothes, and using broad‐spectrum sunscreens.

Besides melanosis, blue light also induces oxidative stress in the skin. A study showed that irradiation of keratinocytes with 41.35 J/cm^2^ of blue light at 453 nm wavelength induced an increase in reactive oxygen species (ROS) as early as within 1 h after exposure.^23^ The mechanisms involve the photoreduction of intracellular flavins and the subsequent production of ROS.^24^, ^25^ These ROS can induce the release of pro‐inflammatory mediators in the skin.^26^ Still, the results among studies are inconsistent and appear to be dependent upon the wavelength used^26^, ^27^, ^28^; whether natural blue light exerts pro‐oxidative and pro‐inflammatory effects remains to be examined. Furthermore, whether antioxidant supplements and sunscreens could prevent those pro‐oxidative and pro‐inflammatory effects in response to blue light should be explored.

This study has limitations. The participants were from a single center, and the sample size was small. Therefore, stratification based on skin phototype could not be performed. Only females were included, and whether males could show similar effects (because of differences in hormones) is unknown. Even if dose equivalence was calculated, the 80 J/cm^2^ dose, for example, was given within a much shorter time than 165 min, which could induce reactions that would not be observed in real life.

## 5 CONCLUSION

In conclusion, blue light at 20 J/cm^2^ induced a smaller change in pigmentation in melasma patients than in healthy women, but the effect of blue light at 40, 60, and 80 J/cm^2^ was similar. The effect of blue light irradiation lasted for 2 weeks in melasma patients and healthy individuals. These results may guide evidence‐based sun protection strategies in patients with melasma and have implications in the context of electronic device use. These results call for enhancing the general population's awareness of blue light and UV protection.

## CONFLICT OF INTEREST STATEMENT

The authors declare no conflict of interest.

## ETHICS STATEMENT

This study was reviewed by the Ethics Committee of the First Affiliated Hospital of Kunming Medical University. Informed consent was obtained from all subjects, and the informed consent form was signed.

## Supporting information

Supporting Information

Supporting Information.

Supporting Information.

Supporting Information.

## Data Availability

All data generated or analyzed during this study are included in this published article/as supplementary information files.
