# Supplementary material for: Impact of blue light on skin pigmentation in patients with melasma
Source: Skin Res Technol. 2023 Jul 2;29(7):e13401. doi: 10.1111/srt.13401 (PMC10315449; doi:10.1111/srt.13401)
Supplement: Supplementary file 1 — Supporting Information [file SRT-29-e13401-s004.docx]

**Supplementary Table S1.** Investigator’s Global Assessment Scale (IGAS)

| Scores | Pigmentation |
| --- | --- |
| 0 | No pigmentation |
| 1 | Barely visible pigmentation |
| 2 | Mild pigmentation |
| 3 | Moderate brown pigmentation |
| 4 | Obvious dark-brown pigmentation |
| 5 | Near black pigmentation |
