# Supplementary figures and images for: Impact of blue light on skin pigmentation in patients with melasma
Source: Skin Res Technol. 2023 Jul 2;29(7):e13401. doi: 10.1111/srt.13401 (PMC10315449; doi:10.1111/srt.13401)

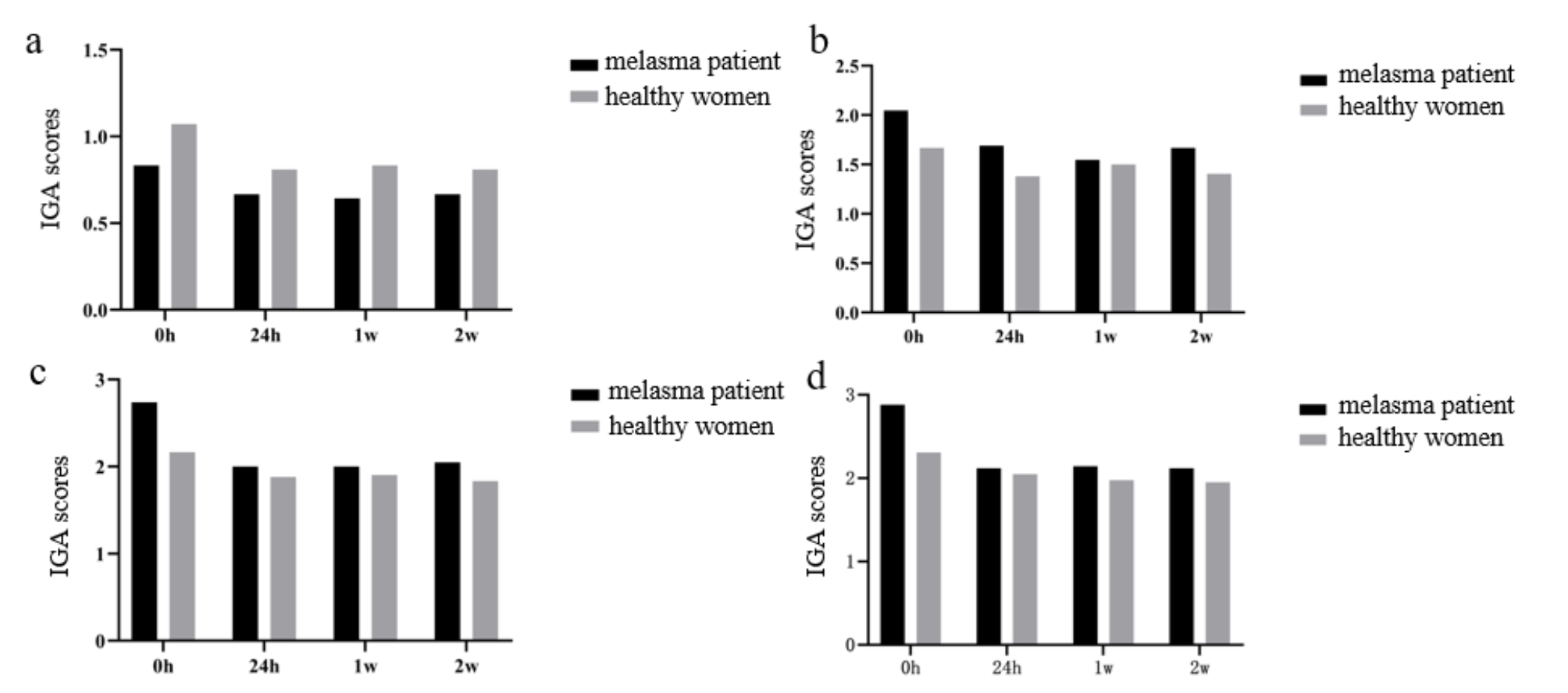

Supplement: Supplementary file 2 — Supporting Information. [file SRT-29-e13401-s003.jpg]

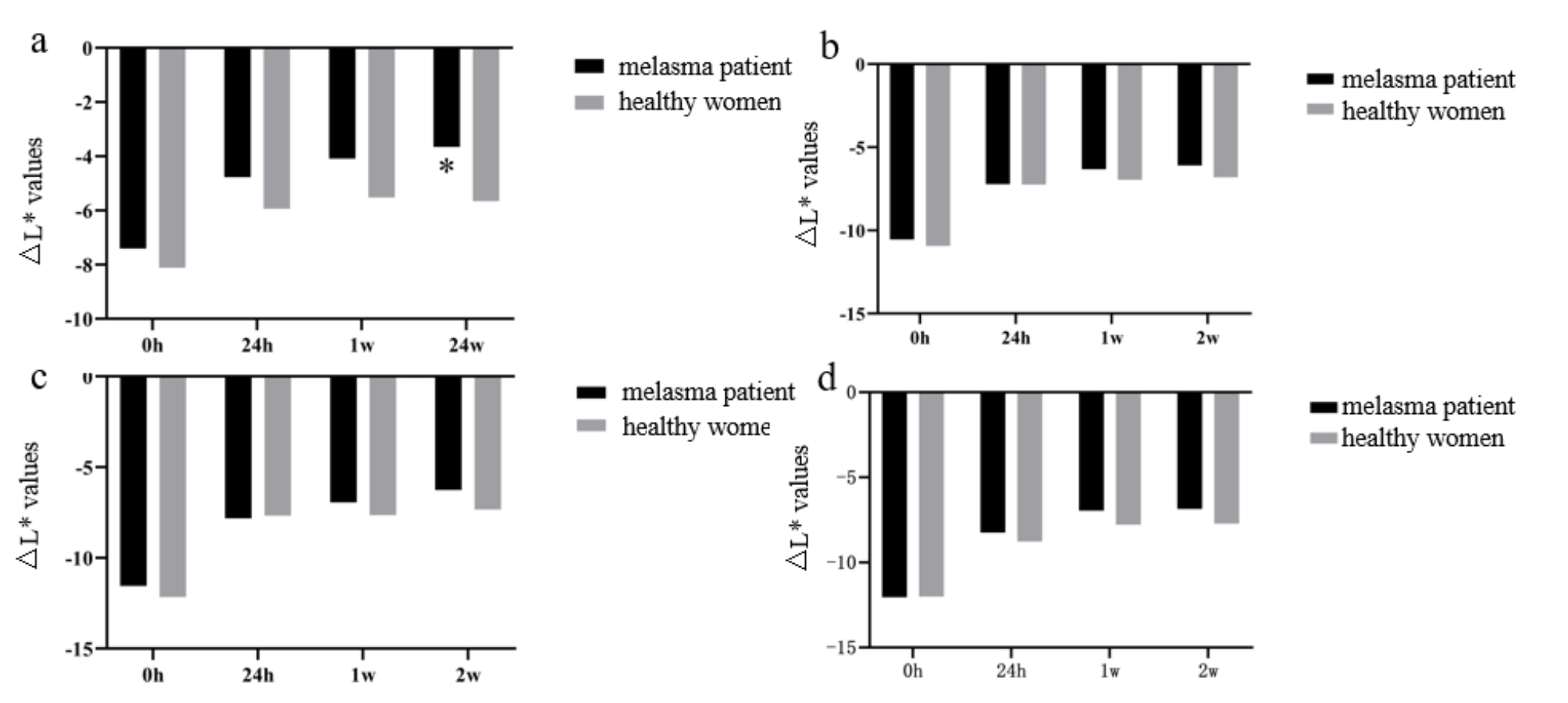

Supplement: Supplementary file 3 — Supporting Information. [file SRT-29-e13401-s001.jpg]

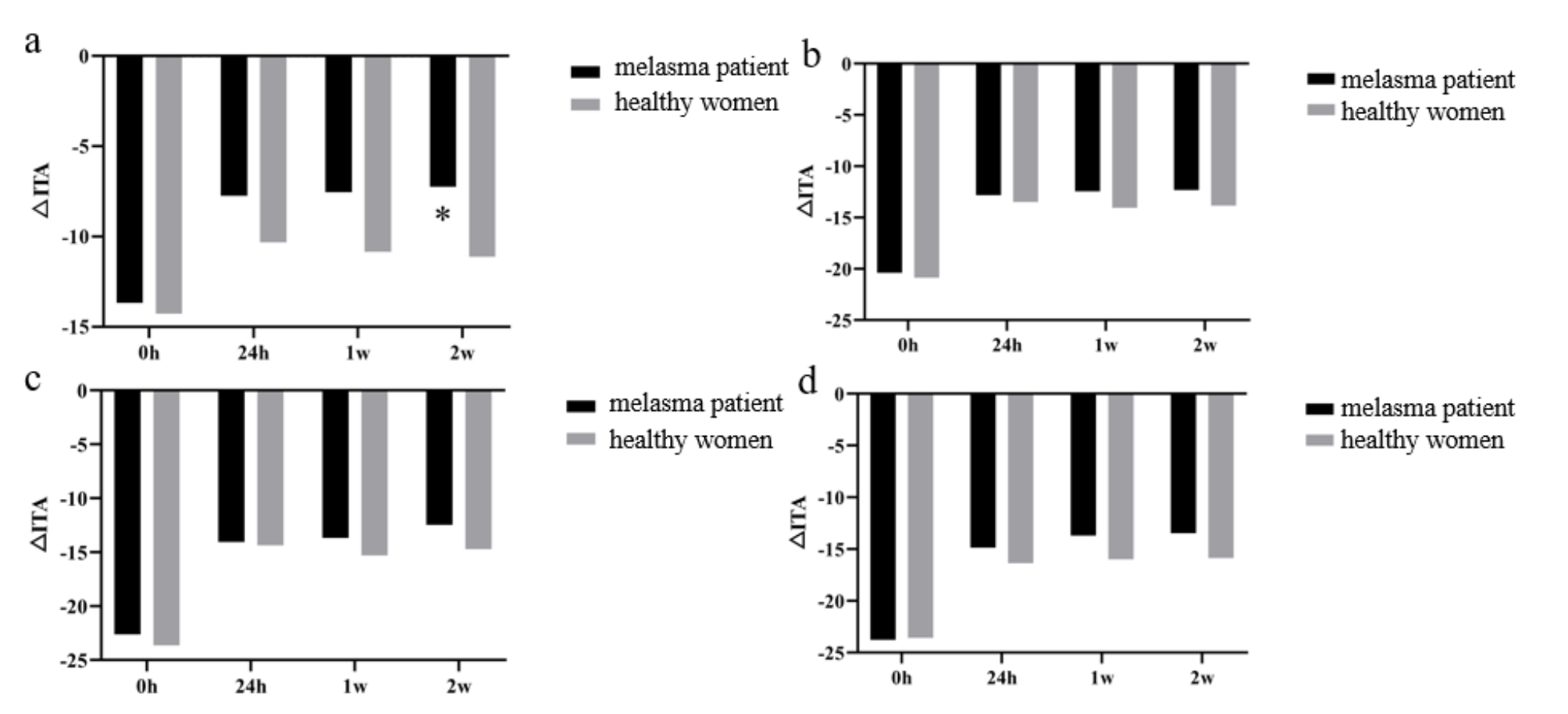

Supplement: Supplementary file 4 — Supporting Information. [file SRT-29-e13401-s002.jpg]
